# A preclinical CT and MRI Liver Imaging Dataset with Anatomical, Functional and Segmentation Data

**DOI:** 10.1038/s41597-026-07003-x

**Published:** 2026-03-23

**Authors:** Sarah Schraven, Catherine Gonzalez, Ferhan Baskaya, Lara Krott, Anika Beckers, Renée Michèle Girbig, Ramona Brück, Diana Möckel, Marie-Luise Berres, Kai Markus Schneider, Angela Schippers, Fabian Kiessling

**Affiliations:** 1 RWTH Aachen University, Institute for Experimental Molecular Imaging, Aachen, Germany; 2 RWTH Aachen University, IT Center, Aachen, Germany; 3 Department of Internal Medicine III, University Hospital RWTH Aachen, Aachen, Germany; 4 Department of Medicine 1, University Hospital Carl Gustav Carus Dresden, TUD Dresden University of Technology, Dresden, Germany; 5 Center for Regenerative Therapies Dresden (CRTD), TUD Dresden University of Technology, Dresden, Germany; 6 Else Kroener Fresenius Center for Digital Health, Medical Faculty Carl Gustav Carus, TUD Dresden University of Technology, Dresden, Germany; 7 Department of Pediatrics, University Hospital RWTH Aachen, Aachen, Germany; 8 Fraunhofer MEVIS, Institute for Medical Image Computing, Aachen, Germany

**Keywords:** Metabolic disorders, Anatomy, Research management, Cancer

## Abstract

Chronic liver diseases (CLD) account for more than 2% of deaths worldwide. Extensive research has been conducted to better understand CLD, generating vast amounts of data. However, only a small fraction of raw preclinical data are publicly available, posing a significant challenge for transparency, reproducibility, and data reuse. Therefore, we built a preclinical liver imaging dataset, the first of its kind to our knowledge. The database contains longitudinal liver MRI scans from mice with hepatocellular carcinoma, metabolic dysfunction-associated steatohepatitis (MASH, formerly NASH), and fibrosis, as well as CT scans of mice with MASH and mice carrying a dysfunctional ICAM-1 gene. Superimposable MRI and CT scans bridge the gap between the modalities. Some of the 222 murine scans have annotated segmentations. Metadata containing both scan and mouse parameters are organized using a tailored metadata profile in ISA-Tabs. This dataset enables advanced image analysis, such as building tools for automated segmentation, train radiomics analysis tools, or can be used as a reference control dataset.

## Background & Summary

Chronic liver diseases (CLD) account for more than 2% of deaths worldwide^1^. The major causes of CLD are viral hepatitis (Hepatitis B virus, Hepatitis C virus) and steatotic liver diseases such as metabolic dysfunction-associated steatotic liver disease and alcohol-associated liver disease, with risk factors including low childhood vaccination rates, exposure to infected blood, obesity, and increased alcohol consumption. In CLD, chronic inflammation and fibrosis can over time lead to liver cirrhosis which has its own complications and consequences such as portal hypertension, hepatocellular insufficiency, and hepatocellular carcinoma^2,3^.

Medical imaging can be used to diagnose and monitor liver cirrhosis as an alternative to invasive biopsies. In the clinic, ultrasound is the most commonly used imaging modality due to its wide availability and low cost^4,5^, but also elastography, CT, and MRI are applied to substantiate and refine the diagnosis^6^. In preclinical investigations, MRI is mainly used due to its high versatility^7,8^. MRI can grade fibrosis and steatosis, besides accurately assessing liver size and detect and differentiate between different types of lesions. In addition, MRI can reduce the need for contrast agents, which are not always well tolerated.

Existing preclinical imaging databases include CT data from healthy mice^9^ or include different types of disease models and imaging modalities, such as the Preclinical Image DAtaset Repository (PIDAR)^10^. In addition, initiatives to create a mouse atlas are helpful to the research community^11^, such as published by Dogdas *et al*.^12^, which is based on a single animal, or Wang *et al*.^13^, who propose a deformable atlas. In contrast to these examples and the liver CT data of Fiebig *et al*.^14^, this dataset focuses on multimodal preclinical liver and whole-body imaging data from CLD mice and healthy controls.

**Fig. 1:**
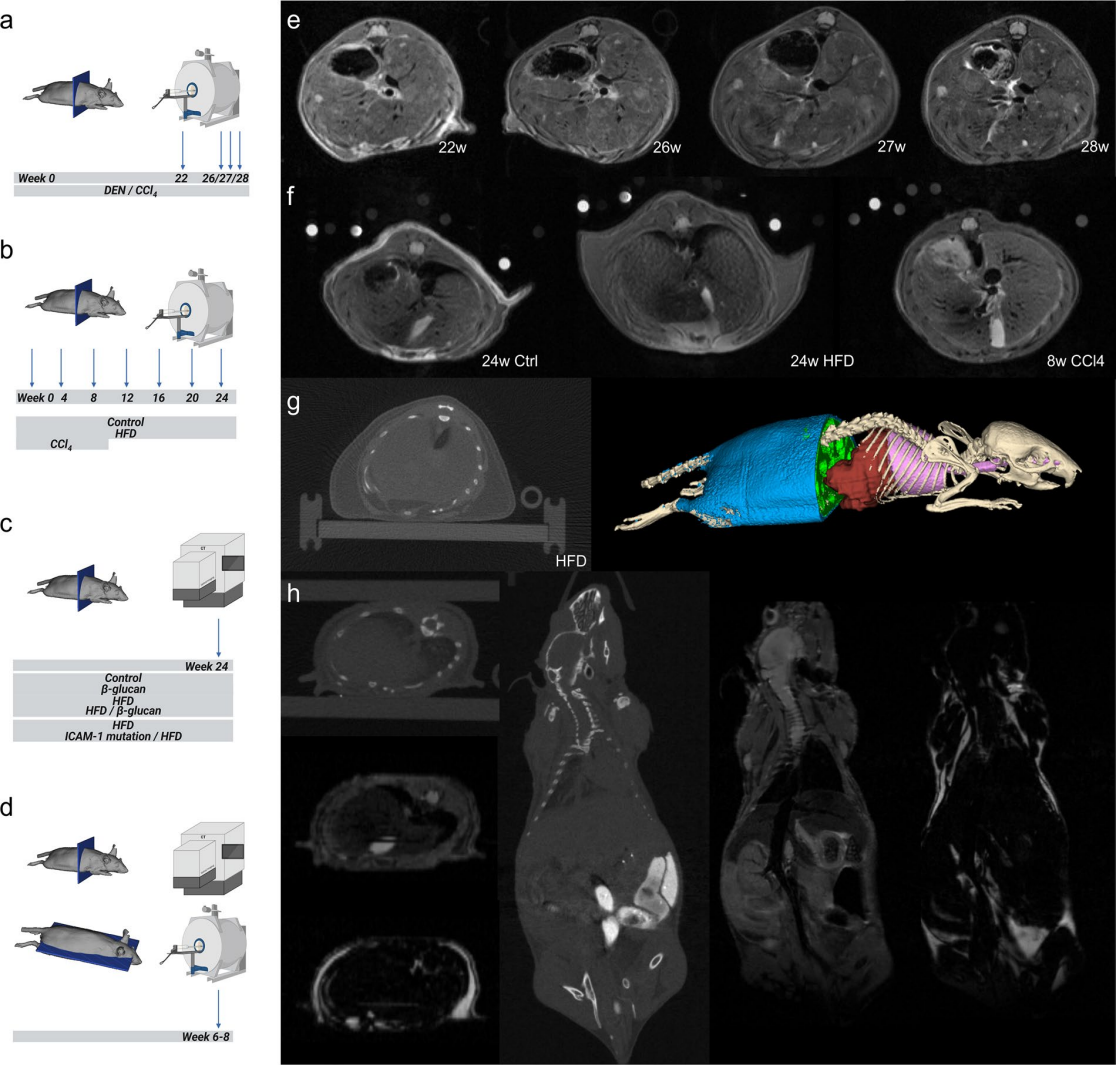
Representative CT and MRI liver scans. In (**a**–**d**) scanning direction and intervals and imaging modalities are shown in addition to study groups. In (**e**) longitudinal native T2w MRI of the liver of an hepatocellular carcinoma model from 22 to 28 weeks is shown, (**f**) displays T1w MRI of healthy, MASH/HFD, and toxic liver injury model (left to right) of the multiparametric MRI study^7^, (**g**) shows CT (left) to image liver size and body fat analysis segmentation (right) of HFD wild-type mice, (**h**) presents whole-body CT and (water and fat) MRI in axial (left) and coronal (right) views of healthy mice^29^. DEN: diethylnitrosamine, HFD: high-fat diet.

To improve the understanding of CLD, scientists have been performing extensive research, resulting in the generation of large data amounts. Out of all the publicly available data, however, only a small fraction of (raw) data is available which remains a major concern. As data availability is necessary for open science and data reuse, we intend to build a dataset of preclinical liver imaging data, which is the first of its type to our knowledge. Via this way, the data can be reused by the community. Possible data reuse strategies are for example, i) training computational models, like generative adversarial networks^15^, ii) creating an attenuation correction atlas for PET by use of overlapping CT and MRI data^16^, iii) training segmentation tools^17^, iv) detecting malignancies or pathological changes by computational models^18^, v) predicting disease progression^19^, and vi) reducing animal numbers by using control animals of one of the studies in case of a similar setup^20^. To give a more concrete example on computational research, livers of healthy and diseased mice might be segmented and analyzed to apply or develop neural networks for segmentation or contour extraction (see Fig. 1 liver segmentation as an example)^21,22^.

## Methods

There are five subsets of studies that have been pooled into one dataset to increase disease spectra and variety of modalities, including (functional) murine MRI data of hepatocellular carcinoma, liver fibrosis and steatosis, as well as whole-body CT data from mice fed with high-fat diet, and combined CT and MRI scans of healthy mice. The latter scans are also provided with segmentations of the liver and other organs. See Table 1 and Fig. 1 for more information on these studies, a study overview, and sample images. In addition, imaging parameters and information on segmentation labels can be found in Tables 1–6, 8–11.

**Table 1:** Scan protocols of studies.

Study	Scan protocol(s)	Study mice and number of scans (per protocol)
**Hepatocellular carcinoma MRI**	- anatomical T2-weighted spin echo (RARE) - diffusion-weighted imaging (EPI)	N = 7 WT HCC, control antibody IgG1 N = 7 WT HCC, control combination antibodies IgG1 + IgG2b 51 scans
**Multiparametric Liver MRI**	- anatomical T1-weighted gradient echo (FcFLASH) - anatomical T2-weighted spin echo (RARE) - apparent diffusion coefficient mapping (EPI) MR1 - T1-weighted gradient echo (w/o fat suppression, (FcFLASH)) - T1 relaxometry (pre and post gadoxetic acid (RARE VTR)) - dynamic contrast enhanced MRI (FLASH) - repetition of anatomical T1-weighted gradient echo (post gadoxetic acid (FcFLASH)) MRI2 - T2 relaxometry (pre and post ferucarbotran (MSME)) - dynamic susceptibility contrast MRI (EPI) - repetition of anatomical T2-weighted spin echo (post ferucarbotran (RARE))	N = 7 C57BL/6 J + HFD N = 10 C57BL/6 J + CCl_4_ N = 7 C57BL/6 J 123 scans
**Beta-glucans liver CT**	- Ultra-focus fast scan mode	N = 3 C57BL/6J N = 3 C57BL/6 J + ß-gluc N = 6 C57BL/6 J + HFD N = 6 C57BL/6 J + HFD & ß-gluc 18 scans
**ICAM-1 CT fat segmentations**	- Ultra-focus fast scan mode	N = 3 C57BL/6 J + HFD N = 4 C57BL/6 J ICAM-1-KO + HFD 7 scans
**CT-MRI whole body**	- Total body normal scan mode - Dixon-based T2-weighted fat-water-separated turbo RARE (rapid acquisition with relaxation enhancement)	N = 12 Crl:SKH1-Hrhr mice 23 scans

More details on MRI sequence parameters and CT scan parameters can be found in Tables 2–6. HCC: Hepatocellular carcinoma, HFD: high-fat diet.

**Table 2:** Detailed imaging parameters of the HCC MRI protocol.

Data type	Details (type of sequence)
anatomical T2-weighted spin echo (RARE)	- FOV, 40 × 40 mm^2^ - matrix, 256 × 256 - effective echo time, 25 ms - repetition time, 2929.08 ms - echo spacing, 8.3 ms - RARE factor 8 - resolution, 156 μm/pixel - in-plane - (axial) slice thickness, 0.5 mm without gaps - fat suppression
diffusion-weighted imaging (EPI)	- FOV, 40 × 40 mm^2^ - matrix, 96 × 96 - effective echo time, 25 ms - repetition time, 5000 ms - echo spacing, 0.64 ms - max. b-value (3915.18 s/mm2) - resolution, 417 μm/pixel - in-plane - (axial) slice thickness, 1 mm with 0.25 mm slice gaps

**Table 3:** Detailed imaging parameters of the Multiparametric Liver MRI protocol.

Data type	Details (type of sequence)
anatomical T1-weighted gradient echo (FcFLASH)	- FOV, 40 × 40 mm^2^ - matrix, 192 × 192 - effective echo time, 3 ms - repetition time, 1 resp. cycle - resolution, 208 μm/pixel - in-plane - (axial) slice thickness, 0.5 mm with 0.25 mm slice gaps - fat suppresion - bandwidth, 75 kHz
anatomical T2-weighted spin echo (RARE)	- FOV, 40 × 40 mm^2^ - matrix, 192 × 192 - effective echo time, 25 ms - repetition time, 3 resp. cycles - echo spacing, 8.3 ms - RARE factor 8 - resolution, 208 μm/pixel - in-plane - (axial) slice thickness, 0.5 mm with 0.25 mm slice gaps - fat suppresion - bandwidth, 72 kHz
apparent diffusion coefficient mapping (EPI)	- FOV, 40 × 40 mm^2^ - matrix, 96 × 96 - effective echo time, 25 ms - repetition time, ≥5000 ms - echo spacing, 0.6 ms - b-values (29, 103, 205, 306, 407, 508, 608, 709, 810, 910, 1010 s/mm^2^) - resolution, 417 μm/pixel - in-plane - (axial) slice thickness, 1 mm with 0.25 mm slice gaps - bandwidth, 150 kHz

General part, before MRI1 and MRI2, which are in Tables 4, 5.

**Table 4:** Detailed imaging parameters of the Multiparametric Liver MRI1 protocol.

Data type	Details (type of sequence)
T1-weighted gradient echo (w/o fat suppression, (FcFLASH))	- see T1wI - water suppression prepulse 1030 Hz - bandwidth, 70 kHz
T1 relaxometry (pre and post gadoxetic acid (RARE VTR))	- FOV, 40 × 40 mm^2^ - matrix, 112 × 112 - effective echo time, 12 ms - repetition times (5500, 3000, 1500, 800, 400, 200 ms) - echo spacing, 6 ms - RARE factor 4 - resolution, 357 μm/pixel - in-plane - (axial) slice thickness, 1 mm with 0.25 mm slice gaps - bandwidth, 75 kHz
dynamic contrast enhanced MRI (FLASH)	- FOV, 40 × 40 mm^2^ - matrix, 128 × 128 - effective echo time, 1296 ms - repetition time, 4.4 ms - resolution, 313 μm/pixel - in-plane - (axial) slice thickness, 1 mm without slice gaps - bandwidth, 200 kHz
repetition of anatomical T1-weighted gradient echo (post gadoxetic acid (FcFLASH))	- see above

General part and MRI2 are in Tables 3, 5, respectively.

**Table 5:** Detailed imaging parameters of the Multiparametric Liver MRI2 protocol.

Data type	Details (type of sequence)
T2 relaxometry (pre and post ferucarbotran (MSME))	- FOV, 40 × 40 mm^2^ - matrix, 144 × 144 - echo times (7, 13, 20, 27, 34, 40, 47, 54, 60, 67, 74, 81, 87, 94, 101, 107, 114, 121, 128, 134, 141, 148, 154, 161, 168 ms) - repetition time, 2000 ms - echo spacing, 6.7 ms - resolution, 278 μm/pixel - in-plane - (axial) slice thickness, 1 mm with 0.25 mm slice gaps - bandwidth, 76 kHz
dynamic susceptibility contrast MRI (EPI)	- FOV, 40 × 40 mm^2^ - matrix, 96 × 96 - effective echo time, 13 ms - repetition time, 1000 ms - echo spacing, 6.7 ms - resolution, 417 μm/pixel - in-plane - (axial) slice thickness, 1 mm without slice gaps - bandwidth, 250 kHz
repetition of anatomical T2-weighted spin echo (post ferucarbotran (RARE))	- see above

General part and MRI1 are in Tables 3, 4, respectively.

**Table 6:** Detailed imaging parameters of all CT studies.

Data type	Details (type of sequence)
**i. Beta-glucans liver CT**	
Ultra-focus fast scan mode	- tube voltage, 55 kV - tube current, 0.17 mA - exposure time of 75 ms
**ICAM-1 CT fat segmentations**	—
Ultra-focus fast scan mode	- tube voltage, 65 kV - tube current, 0.13 mA - exposure time of 75 ms
**ii. CT-MRI whole body**	
Total body normal scan mode	- tube voltage, 55 kV - tube current, 0.17 mA
Dixon-based T2-weighted fat-water–separated turbo RARE (rapid acquisition with relaxation enhancement)	- FOV, 80 × 55 × 17.6 mm^3^ - matrix, 384 (frequency - encoding direction) × 264 (phase encoding direction) × 44 (slices) - repetition time, 2715.0 ms - effective echo time, 22.6 ms - echo spacing, 11.3 msdata - RARE factor 4 - echo shift for water-fat separation, 0.37 ms - resolution, 208 μm/pixel - in-plane (coronal) slice thickness, 400 μm without slice gaps - receiver bandwidth, 78,125.0 Hz

### MRI assessment of hepatocellular carcinoma progression

To study the dynamics of the cellular immune compartment within the tumor microenvironment of a chronically inflamed and damaged liver during both progression and regression of HCC, the toxin-based DEN/CCl₄ model was employed^23,24^. The fibrosis-associated HCC mouse model relies on the induction of spontaneous tumor formation through a single administration of the carcinogen diethylnitrosamine (DEN) followed by repeated injections of carbon tetrachloride (CCl₄). The single dose of the tumor initiator DEN was administered to C57BL/6 J mice (WT, RWTH Aachen University) 14 days after birth at a concentration of 25 mg/kg body weight (BW) (diluted in PBS). At 4 weeks of age, the animals also received CCl_4_ injections (0.5 ml/kg BW, diluted in germ oil) once a week over a period of 22 weeks. Both agents were applied by i.p. injection. Following the manifestation of HCC at 26 weeks of age, a combined therapy consisting of immune checkpoint blockade and anti-angiogenic therapy was implemented to induce tumor regression. The efficacy of the therapeutic approach in promoting HCC regression was assessed by comparing treated groups with control cohorts receiving isotype antibodies. Isotype antibodies utilized in the study were IgG1 (BioXcell, BE0088; 40 mg/kg BW) and IgG2b (BioXcell, BE0090; 200 µg/mouse). Control groups received either IgG1 isotype antibody alone (WT, *n* = 7) or a combination of both isotypes (WT, *n* = 7). The first control group received 6 i.p. injections of IgG1 over two weeks, while the second control group received 5 i.p. injections of the combined antibodies over the same time frame. Please note that as this study has not yet been published, only data from control animals are included in the dataset.

To monitor tumor growth and size during HCC progression and regression, MRI was performed using a 7 T Biospec 70/20 scanner (Bruker BioSpin, Ettlingen, Germany) with an RF RES 300 1H 075/040 QSN TR volume coil (T13161V3), at (22,) 26, 27, and 28 weeks of age under isoflurane anesthesia. To obtain structural information about the liver, a T2-weighted sequence was used. Additionally, to assess Brownian motion and thus extracellular matrix composition and cellularity within the liver tissue, a diffusion-weighted sequence was included (scan parameters are shown in Table 2). Both sequences were respiration-triggered to minimize breathing artifacts.

### Multiparametric MRI to assess hepatic fibrosis and steatosis

The objective of Baskaya *et al*.^7^ was to explore the feasibility of CLD classification using longitudinal multiparametric MRI. For this purpose, 8–10-week-old male C57BL/6 J wild-type mice (Janvier Labs, Le Genest‐Saint‐Isle, France) were randomly assigned to two distinct CLD models, a high-fat diet (HFD) group (*n* = 7) and a CCl_4_ group (*n* = 10), as well as an untreated control group (*n* = 7). To induce metabolic dysfunction associated steatohepatitis (MASH), mice were fed with HFD chow containing 40% fat, 20% fructose, and 2% cholesterol (Research Diets Inc, New Brunswick, NJ) for 24 weeks. In the CCl_4_ group, inflammation-induced fibrotic remodeling of the liver was induced by i.p. injections of CCl_4_ (diluted in corn oil, Sigma Aldrich) at a dose of 0.6 ml/kg BW twice a week for 8 weeks. In this group, four out of ten mice died. The untreated control group was fed with standard chow (ssniff, Soest, Germany) for 24 weeks.

MRI was performed using a 7 T Biospec 70/20 scanner (Bruker BioSpin, Ettlingen, Germany) with an RF RES 300 1H 075/040 QSN TR volume coil (T13161V3). Anatomical and functional MRI measurements under isoflurane anesthesia were conducted at 0, 4, 8, 12, 16, 20, and 24 weeks in the HFD and control groups, and at 0, 4, and 8 weeks in the CCl_4_ group (at least two days post CCl_4_ injection). For organizational reasons, the time points in the database must be read as time points after disease induction and not as injection time points, therefore, 0, 4, 8, 12, 16, 20, and 24 weeks correspond to 0, 40320, 80640, 120960, 161280, 201600, and 241920 min, respectively.

To avoid interference between the two contrast agents gadoxetic acid (Primovist, Bayer, Germany) and ferucarbotran (VivoTrax™, Magnetic Insight Inc., Alameda, USA), two separate MRI protocols, labeled MR1 and MR2, were performed following a 48-hour washout period (see Tables 3–5). The first contrast agent was used to evaluate hepatocyte function, while the latter was used to assess macrophage function. As respiration can cause artifacts in MRI, all liver scans were acquired with respiration gating. In both the MR1 and MR2 protocols, liver anatomy was assessed by T1-weighted gradient echo and T2-weighted spin echo sequences (with fat suppression). Detailed structural information at the cellular level and extracellular matrix composition was obtained by apparent diffusion coefficient mapping using a diffusion-weighted echo planar imaging sequence. Lower apparent diffusion coefficients correlated with fibrosis. Structural imaging was followed by functional sequences to assess hepatocyte function (MR1), liver damage (MR2), and macrophage activity (MR2).

In the MR1 protocol, a fat-selective T1-weighted gradient echo sequence with water suppression was acquired to determine the liver fat content. Paraffin oil-filled tubes were placed in the field of view for reference. T1 relaxometry was performed before and after gadoxetic acid injection (0.025 mmol/kg BW in 100 μL 0.9% saline). Between the relaxometry mapping, the hepatocyte function was assessed, and the contrast agent was injected manually at approximately 10 µL/s, a dynamic contrast enhanced MRI scan of a central slice in the liver (1600 acquisitions) was performed after 30 s of baseline scans. Lower gadoxetic acid uptake correlated with reduced hepatocyte function. Finally, the anatomical T1-weighted gradient echo sequence was acquired again, but this time contrast-enhanced. The MR1 protocol took 50–55 min to complete.

The MR2 protocol continued with T2 relaxometry mapping before and after ferucarbotran injection (8 μmol Fe/kg BW ferucarbotran in 100 μL 0.9% saline) using a multi-slice multi-echo T2 mapping sequence. Low R2 values correlated with higher liver damage demonstrated by serum glutamic-oxaloacetic transaminase levels. To assess macrophage phagocytic activity, a dynamic susceptibility contrast MRI was performed between these scans, with contrast agent injected at 10 µL/s after 30 s of dummy scans. Lower ferucarbotran uptake indicated decreased macrophage function. The MR2 protocol ended with a contrast-enhanced T2-weighted spin echo sequence (as before). The MR2 acquisition time was completed after 40–45 min.

### Liver size measurements by native CT to test the effect of beta-glucans on steatosis

To study the benefit of oat beta-glucan in CLD, more specific, metabolic dysfunction-associated steatotic liver disease, amongst others, liver volume and fat distribution were measured by µCT measurements^25^. Hence, male 8 weeks old C57BL/6 J mice (Janvier labs, Le Genest-Saint-Isle, France) were fed with a chow diet (control, *n* = 3) or with a western style high fat diet containing 40% fat, 20% fructose, and 2% cholesterol (Research Diets Inc, New Brunswick, NJ, HFD, *n* = 6) for 24 weeks, as described in Jaeger *et al*.^25^. Solubilized oat beta-glucan (Garuda, Exeter, USA) was administered with the drinking water (control + beta-glucan, *n* = 3; HFD + beta-glucan, *n* = 6). The body fat distribution was assessed under isoflurane anesthesia with a total body normal scan in a hybrid micro-CT optical imaging system (MILabs B.V., Houten, the Netherlands) with an X-ray tube voltage of 55 kV, tube current of 0.17 mA, an isotropic voxel size of 140 μm, and a scan time of 4 minutes and 11 seconds (Table 6). To analyze fat composition, an interactive segmentation protocol was loaded and livers were segmented manually for volume measurements (Imalytics Preclinical, Gremse-IT GmbH, Aachen, Germany)^26^.

### Liver size measurements by native CT to test the role of ICAM-1

To assess the role of intercellular adhesion molecule 1 (ICAM-1, or CD54), a cell surface protein with a role in inflammation and immunity^27^, in metabolic dysfunction, WT mice and mice with an ICAM-1 mutation were studied in their fat distribution and liver size^28^. Therefore, 8–12-week-old male WT (C57BL/6 J, RWTH Aachen University) and ICAM-1 mutant mice (B6.129S7-*Icam1*^*tm1Bay*^/J, RWTH Aachen University)^27^ were fed a HFD (40% fat, 20% fructose, 2% cholesterol, Brogaarden, Lynge, Denmark) for 12 weeks. µCT scans were performed under isoflurane anesthesia in ultra-focus fast mode in a hybrid micro-CT optical imaging system (MILabs B.V., Houten, the Netherlands) with a tube voltage of 65 kV, a tube current of 0.13 mA, an isotropic voxel size of 140 μm, and a scan time of 37 s (Table 6). Segmentations (Imalytics Preclinical, Gremse-IT GmbH, Aachen, Germany)^26^ of body fat (subcutaneous and visceral), liver, bones, and the lungs are included.

### Whole-body CT-MRI of healthy mice

This study tracked the biodistribution of immunoglobulins^29^, therefore healthy female Crl:SKH1-Hrhr nude mice (Charles River Laboratories, Wilmington, MA) of 6–8 weeks were imaged directly after injection as well as 4.5 h after injection of fluorescently labeled immunoglobulins. However, the optical fluorescence data are not included in the dataset as it is outside the scope of the latter. Imaging was performed under isoflurane anesthesia at the two time points inside the hybrid imaging animal holder (MILabs B.V., Houten, the Netherlands) first in the hybrid micro-CT optical imaging system (MILabs B.V.), then the MRI scan was performed. First the optical fluorescence imaging was performed, then the mouse holder was moved to the CT unit, and mice were scanned with the total body normal protocol with tube voltage of 55 kV, tube current of 0.17 mA, an isotropic voxel size of 140 μm (Table 6). Then, mice kept inside the animal holder were transferred to perform MRI in a 7 T BioSpec 70/20 USR MRI scanner (Bruker BioSpin, Ettlingen, Germany) equipped with an RF Res 300 1H 112/086 QSN TO AD volume coil (T12053V3). For whole-body imaging a Dixon-based T2-weighted fat-water-separated turbo RARE (rapid acquisition with relaxation enhancement) with the in Table 1 specified parameters was performed. During fluorescence tomography reconstructions, the vaseline-filled inner marker holes in the animal holder enabled overlap of CT and MRI data. For CT and MRI scans, segmentations of bladder, bone (only CT), brain (only MRI), caecum, colon, heart, intestine, kidney, liver, lungs, spinal cord (only MRI), spleen, and stomach are included. Fluorescence tomography data are not included in the dataset.

### Animal housing

All experiments were approved by the North Rhine-Westphalia ‘Stage agency for nature, environment, and consumer protection (Landesamt für Natur-, Umwelt- und Verbraucherschutz Nordrhein-Westfalen, LANUV). Mice were socially housed with a conventional light cycle of 12 hours (7 am to 7 pm), at 20–24 °C, humidity of 45–65% and bedding was changed weekly. All animals were kept in sterile, individually ventilated cages. Sterilized drinking water and (standard) chow were available to the mice *ad libitum*.

## Data Records

The dataset is available at Zenodo^30^, where the data are stored together with the metadata in ISA-Tabs as specified in the metadata profile, which is shown in Tables 7, 8, 9, 10, 11. In the ISA-Tabs, file names are linked with all the relevant metadata related to the mice and imaging modalities used. The same metadata as displayed in the assay files (a_*.text) can also be found on the research data management platform Coscine, which is accessible upon request.

**Table 7:** General metadata profile.

Field name	Field type	Additional information
**Creator**	- String - Required	
**Study ID**	- String - Required	A sequence of characters used to identify, name, or characterize the study.
**Animal ID**	- String - Required	Includes the animal number
**Animal strain**	- String	Breed of animal (e.g. for breed of mouse the strain may be, C57BL/6, SKH1, BALB/c)
**Supplier**	- String	
**Image modality**	- List - Required	Type of device, process or method used to acquire or derive data. CT, MRI, MRI-CT, or US.
**Device name**	- String	
**Brand**	- List - Required	Includes a list of various brands: Bioemtech, Bruker, Fuji Film Visualsonics, Mediso, MILabs, Molecubes, MR Solutions, PerkinElmer, Spectral Instruments Imaging, TriFoil Imaging, or other
**Coil**	- String	When MRI is chosen. The classification of the coil that is used in a magnetic resonance imaging procedure. Usually refers to the anatomical location for where the coil is placed such as head, body or breast. (e.g. RF Res 300 1H 112/086 QSN TO AD volume coil)
**Source**	- List	Contrast-enhanced or native
**Data format**	- String	
**Data type**	- List	Raw data or reconstructed data
**Contrast agent name**	- String	
**Longitudinal data**	- Boolean - Required	When multiple time points were measured.
**Time point**	- Integer	In minutes. Time after injection.
**Health status**	- List - Required	Healthy or nor healthy
**Disease model**	- String	
**Age**	- Integer	In days. How long something has existed; elapsed time since birth.
**Sex**	- List	Female or male
**Organ Segmentation**	- Can be added multiple times - For each organ separately - Can also indicate diseased status of organ without segmentation	- Sub metadata profile
**CT imaging parameters**		- Sub metadata profile
**MRI imaging parameters**		- Sub metadata profile

This part describes metadata about the study settings.

**Table 8:** Metadata profile on organ segmentation.

Field	Field type	Additional information
**Organ segmentation**	- List	Available or not available
**Organ**	- List	List of various organs: bone, bladder, brain, caecum, colon, gall bladder, heart, lung, kidney, liver, spine, stomach, thyroid gland, tumor, spleen, small intestine, pancreas, ovaries, uterus, testes, muscle, fat, or other
**Method of segmentation**	- List	Automatically, manual, or semi-automatically
**Affected Organs**	- Boolean	Might the disease model affect the organs’ morphology?

This profile describes from which organs segmentations are available and if the disease model might affect the organs’ morphology.

**Table 9:** Segmentation labels.

Label	Description	Ontology reference
Bladder	Urinary bladder; A membranous sac in many vertebrates that serves for the temporary retention of urine and discharges by the urethra.	http://purl.obolibrary.org/obo/BTO_0001418
Bone	The hard form of connective tissue that constitutes the majority of the skeleton of most vertebrates.	http://purl.obolibrary.org/obo/BTO_0000140
Brain	The portion of the vertebrate central nervous system that constitutes the organ of thought and neural coordination, includes all the higher nervous centers receiving stimuli from the sense organs and interpreting and correlating them to formulate the motor impulses, is made up of neurons and supporting and nutritive structures, is enclosed within the skull, and is continuous with the spinal cord through the foramen magnum.	http://purl.obolibrary.org/obo/BTO_0000142
Caecum	The first part of the large intestine, forming a dilated pouch into which open the ileum, colon, and appendix vermiformis.	http://purl.obolibrary.org/obo/BTO_0000166
Colon	The part of the large intestine that extends from the cecum to the rectum.	http://purl.obolibrary.org/obo/BTO_0000269
Fat	Connective tissue in which fat is stored and which has the cells distended by droplets of fat.	http://purl.obolibrary.org/obo/BTO_0001487
Gall bladder	A small, pear-shaped muscular sac, located under the right lobe of the liver, in which bile secreted by the liver is stored until needed by the body for digestion.	http://purl.obolibrary.org/obo/BTO_0000493
Heart	A hollow muscular organ of vertebrate animals that by its rhythmic contraction acts as a force pump maintaining the blood circulation.	http://purl.obolibrary.org/obo/BTO_0000562
HeartLung	Combination of heart and lungs.	
Intestine	The tubular part of the alimentary canal that extends from the stomach to the anus.	http://purl.obolibrary.org/obo/BTO_0000648
Kidney	One of a pair of vertebrate organs situated in the body cavity near the spinal column that excrete waste products of metabolism, in humans are bean-shaped organs lying behind the peritoneum in a mass of fatty tissue, and consist chiefly of nephrons by which urine is secreted, collected, and discharged into a main cavity whence it is conveyed by the ureter to the bladder.	http://purl.obolibrary.org/obo/BTO_0000671
Liver	A large very vascular glandular organ of vertebrates that secretes bile and causes important changes in many of the substances contained in the blood (as by converting sugars into glycogen which it stores up until required and by forming urea).	http://purl.obolibrary.org/obo/BTO_0000759
Lung	One of the usually paired compound saccular thoracic organs that constitute the basic respiratory organ of air-breathing vertebrates.	http://purl.obolibrary.org/obo/BTO_0000763
Spine	Spinal cord; Body region belonging to the nervous system in the vertebral column.	http://snomed.info/id/12958003
Spleen	A highly vascular ductless organ that is located in the left abdominal region near the stomach or intestine of most vertebrates and is concerned with final destruction of red blood cells, filtration and storage of blood, and production of lymphocytes.	http://purl.obolibrary.org/obo/BTO_0001281
Stomach	A dilatation of the alimentary canal of a vertebrate communicating anteriorly with the esophagus and posteriorly with the duodenum.	http://purl.obolibrary.org/obo/BTO_0001307
Thyroid	A two-lobed endocrine gland found in all vertebrates, located in front of and on either side of the trachea, and producing various hormones, such as triiodothyronine and calcitonin.	http://purl.obolibrary.org/obo/BTO_0001379
Visceral fat	Adipose tissue located inside the peritoneal cavity, packed in between internal organs and torso.	http://purl.obolibrary.org/obo/BTO_0004041

The segmentation labels are presented here together with a description of which label corresponds to which organ.

**Table 10:** Metadata profile for CT imaging parameters.

Field	Field type	Additional information
**Voxel Size X [mm]**	- Decimal	For resolution and image size
**Voxel Size Y [mm]**	- Decimal	
**Voxel Size Z [mm]**	- Decimal	
**Scan angle**	- Decimal	For image quality and X-ray dose
**Angle step degree**	- Decimal	For image quality and X-ray dose
**Exposure [ms]**	- Decimal	For image quality and X-ray dose
**Xray voltage [kV]**	- Decimal	For image quality and X-ray dose
**Xray current [mA]**	- Decimal	For image quality and X-ray dose
**Hounsfield unit calibration**	- Decimal	
**FOV X [mm]**	- Decimal	For resolution and image size
**FOV Y [mm]**	- Decimal	
**FOV Z [mm]**	- Decimal	

The settings for CT scans are captured in this profile.

**Table 11:** Metadata profile for MRI imaging parameters.

Field	Field type	Additional information
**Repetition time**	- Decimal	In milliseconds
**Echo time**	- Decimal	In milliseconds
**Echo spacing**	- Decimal	In milliseconds
**Echo Train Length**	- Integer	RARE factor (Rapid Acquisition Relaxation Enhanced equals FastSpinEcho or TurboSpinEcho) = Echo Train Length
**Slice thickness**	- Decimal	In µm
**Slice gap**	- Integer	In µm
**Matrix X**	- Integer	For resolution and image size
**Matrix Y**	- Integer	
**Matrix Z**	- Integer	
**Respiratory gating**	- Boolean	Triggering measurements by adjusting respiratory gates will increase image quality
**Scan time**	- Integer	In seconds
**FOV X [mm]**	- Decimal	For resolution and image size
**FOV Y [mm]**	- Decimal	
**FOV Z [mm]**	- Decimal	
**Water Suppression**	- Boolean	
**Water suppression prepulse**	- Decimal	Measured in Hertz [Hz]
**Fat Suppression**	- Boolean	
**Repetitions**	- Integer	
**Flip Angle**	- Decimal	
**Averages**	- Integer	
**Pixel Size X**	- Decimal	[μm/Pixel], for resolution and image size
**Pixel Size Y**	- Decimal	[μm/Pixel]

The settings for CT scans are included in this profile.

The file structure is flat. Studies were named as follows and can be searched in the search bar: i) hepatocellular carcinoma MRI, ii) multiparametric liver MRI, iii) beta-glucans liver CT, iv) ICAM-1 CT fat segmentations, and v) CT-MRI whole body. 3D imaging data are saved as.dcm or.nii files, files from iii)-v) are accompanied by 3D segmentations in.seg or.segff files (see study descriptions).

## Data Overview

This dataset contains data from 5 studies, namely, i) a study to monitor HCC development with MRI, ii) multiparametric MRI to evaluate hepatic fibrosis and steatosis with various structural and functional scans, iii/iv) liver volume measurements with CT to investigate the interplay of beta-glucans or ICAM-1 with a HFD including body fat and liver segmentations, and v) whole-body MRI and CT including segmentations of healthy mice.

## Technical Validation

DEN and CCl₄ are commonly used agents to induce HCC, as demonstrated in previous studies through histological analysis^23,24^. In this study, the livers exhibited multiple HCC lesions on MRI, which were further confirmed by the presence of tumors macroscopically observable upon excision. In the secondly described study, by Baskaya *et al*.^7^, MASH and liver fibrosis progression were confirmed by histology. Similarly, Jaeger *et al*.^25^ display by liver histology the effects of beta-glucan and high fat diet, which are in line with the imaging results.

The mice with the dysfunctional ICAM-1 gene used in the study by Eswaran *et al*.^28^ were originally described in Sligh *et al*.^27^ and it was reported that these mice only showed residual membrane-bound ICAM-1 in thymus, lung, and spleen tissues on staining, but none in gut and liver. Before the animals were used in experiments, they were tested for the presence of the mutation by polymerase chain reaction with primers for ICAM-17 (5′CTGAGCCAGCTGGAGGTCTCG3′, ICAM-18 (5′GAGCGGCAGAGCAAAAGAAGC3′), and ICAM-19 (5′AGGACAGCAAGGGGGAGGATT3′), as described in Eswaran *et al*.^28^. All WT and B6.129S7-*Icam1*^*tm1Bay*^/J mice used were healthy and came from the same barrier.

In the combined CT-MRI study^29^, healthy animals were used and CT- and MRI-based segmentations were validated with dice score analyses.

## Data Availability

The dataset is available at 10.5281/ZENODO.17130082.

## Code availability

No custom code has been used to curate the dataset. The data can be downloaded directly without the need for any specific software.
